# Targeting sphingosine-1-phosphate for cancer therapy

**DOI:** 10.1038/sj.bjc.6603400

**Published:** 2006-10-03

**Authors:** R A Sabbadini

**Affiliations:** 1 Department of Biology San Diego State University and Lpath Inc., 5500 Campanile Dr, San Diego 92182-4614, CA, USA

**Keywords:** tumour microenvironment, bioactive lipid signaling, sphingosine-1-phosphate, lipodomics, angiogenisis

## Abstract

This review summarises some important new findings that implicate sphingosine-1-phosphate (S1P) as a potent tumorigenic and angiogenic agent released from cancerous tumours into the tumour microenvironment. Also explored is the novel concept that bioactive lipid signalling molecules, like S1P, can themselves be targets for rational drug design, thereby opening up an entire class of lipidomic-based therapeutics for oncology and other human diseases.

## SPHINGOSINE-1-PHOSPHATE, A BIOACTIVE LIPID WITH TUMORIGENIC EFFECTS

Until recently, phospholipids and their derivatives were considered ‘neutral’ in that they were thought either to serve a simple structural role in cell membrane organisation or to provide energy for beta oxidation, glycolysis and other metabolic processes. It has only recently been appreciated that many lipids have significant roles as bioactive signalling mediators. Good examples of such bioactive lipids include: (i) the eicosanoids (such as the cannabinoids and the leucotrienes), (ii) phospholipids and their derivatives such as phosphatidic acid and platelet activating factor, (iii) lysophospholipids such as lysophosphatidyl choline and lysophosphatidic acid (LPA) and (iv) certain sphingolipids.

**Figure 1:**
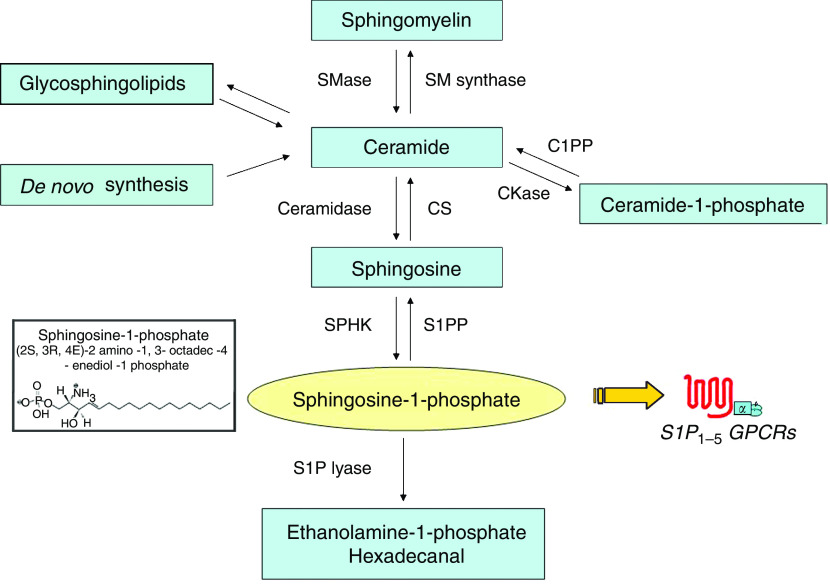
The extracellular signalling mediator, S1P, is produced by phosphorylation of SPH by SPHK. Sphingosine-1-phosphate exerts many of its tumorigenic and angiogenic effects by acting as a ligand for the five GPCRs for S1P (S1P_1–5_). Sphingosine-1-phosphate is irreversibly metabolised to the physiologically inert metabolites, ethanolamine-1-phosphate and hexadecanal. Sphingosine-1-phosphate can also be dephosphorylated by S1P phosphatases (S1PP). Other key enzymes in the cascade are: sphingomyelinase (SMase), which produces CER and choline (not shown); ceramidase, which de-esterifies CER to produce SPH (with a fatty acid product not shown); SM synthase; ceramide kinase (CKase); and ceramide-1-phosphate phosphatase (C1PP).

Sphingolipid signalling mediators represent a group of extracellular and intracellular signalling molecules with pleiotropic effects on important cellular processes, including cancer (Ogretmen and Hannun, 2004). Figure 1 shows the key elements of the sphingolipid signalling cascade, including the bioactive-lipid mediators, ceramide (CER), ceramide-1-phosphate (C1P), sphingosine (SPH) and sphingosine-1-phosphate (S1P). These mediators are derived from sphingomyelin, which is present in the plasma membranes of all mammalian cells.

Although much attention has been paid to S1P as a pleiotropic mediator required for normal embryonic development, particularly for cardiogenesis, vasculogenesis and angiogenesis (Liu *et al*, 2000), S1P has only recently been appreciated for its roles in lymphocyte trafficking, inflammation, cardiovascular and neurological disorders and tumour biology (Hla, 2004; Gardell *et al*, 2006). The major source of S1P is that produced from SPH through the action of sphingosine kinase (SPHK) (Taha *et al*, 2006). The pleiotropic biological activities of S1P are mediated via a family of G protein-coupled receptors (GPCRs) originally known as endothelial differentiation genes (EDG). Five GPCRs have been identified as high-affinity S1P receptors (S1PRs): S1P_1_/EDG-1, S1P_2_/EDG-5, S1P_3_/EDG-3, S1P_4_/EDG-6 and S1P_5_/EDG-8 only identified as late as 1998 (Lee *et al*, 1998). Many responses evoked by S1P are coupled to different heterotrimeric G proteins (G_q−_, G_i_, G_12−13_) and the small GTPases of the Rho family (Gardell *et al*, 2006).

In the adult, S1P is released from platelets (Murata *et al*, 2000) and mast cells to create a local pulse of free S1P (sufficient enough to exceed the *K*_d_ of the S1PRs) for promoting wound healing and participating in the inflammatory response. Under normal conditions, the total S1P in the plasma is quite high (300–500 nM); however, it has been hypothesised that most of the S1P may be ‘buffered’ by serum proteins, particularly lipoproteins (e.g., HDL>LDL>VLDL) and albumin, so that the bio-available S1P (or the free fraction of S1P) is not sufficient to appreciably activate S1PRs (Murata *et al*, 2000). If this were not the case, inappropriate angiogenesis and inflammation would result.

**Figure 2:**
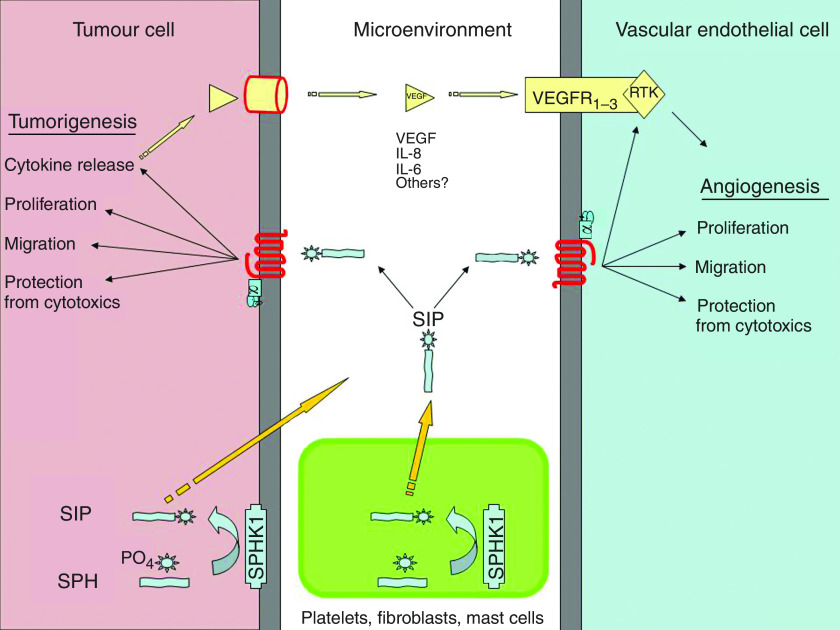
S1P is a tumorigenic and angiogenic growth factor produced normally by blood platelets, mast cells and possibly fibroblasts in the tumour microenvironment. Importantly, cancerous tumour cells upregulate the expression of SPHK1, which may greatly contribute to the putative increased levels of S1P in the tumour microenvironment. The released S1P is able to act in an autocrine or paracrine manner on tumour cells (TCs) and vascular ECs to: (1) promote DNA synthesis and resulting proliferation of both, (2) stimulate migration of cells enhancing the metastatic potential of TCs whereas promoting EC-based angiogenesis and (3) protect TCs and ECs from proapoptotic chemotherapeutic agents. As an additional indirect angiogenic effect, S1P is responsible for the release of proangiogenic growth factors (VEFG, IL-6 and IL-8) from TCs. The combined tumorigenic and angiogenic effects of S1P make it an excellent target for anticancer therapy.

As early as the 1860s, Rudolf Virchow proposed that cancerous tumours occur at sites of chronic inflammation. In 1986, HF Dvorak speculated that tumours are ‘wounds that do not heal’ (Dvorak, 1986). One could argue that the ability of cancer cells to release S1P into the tumour microenvironment is consistent with the idea that cancerous tumours release mediators to trick the body into thinking that it has a wound that needs the infiltration of platelets, fibroblasts, mast cells and neutrophils for the purpose of creating an inflammatory response. The infiltrating cells promote further release of S1P into the tumour microenvironment with the resulting manifestation of the tumorigenic and proangiogenic effects of S1P (Figure 2).

## SPHINGOSINE-1-PHOSPHATE IS TUMORIGENIC

It has been suggested that the balance between CER/SPH levels *vs* S1P provides a rheostat mechanism that decides whether a cell is sent into the death pathway (via CER and/or SPH) or is protected from apoptosis by S1P. One of the key regulatory enzymes of the sphingolipid rheostat mechanism is SPHK, whose role is to convert the death-promoting sphingolipids, CER and SPH, into the growth-promoting S1P. In most, but not all of the cell types tested, S1P promotes cell proliferation and is involved in chemotaxis and cytoskeletal rearrangement via the Rho signalling system.

Cancer cells are notorious for being opportunistic in co-opting key signalling systems. As such, they take advantage of the sphingolipid rheostat by promoting conditions that favour the production of S1P. Many cancer cells accomplish this through an upregulation of the expression of one isoform of the kinase, SPHK1, the isoform that is thought to be responsible for S1P release into the extracellular compartment. This raises the possibility that *sphk1* is an oncogene and, as a result, directs attention to the kinase as a protein target for anticancer drug discovery (Milstien and Spiegel, 2006).

The first studies proposing *sphk1* as an oncogene observed that NIH-3T3 fibroblasts stably transfected with the SPHK1 exhibited enhanced cell proliferation accompanied by increased S1P production (Vadas and Gamble, 1996). Cells overexpressing SPHK1 escaped contact inhibition, a property commonly exhibited by transformed cells. This observation is consistent with reports showing that S1P enhances metastatic potential of human cancer cell lines by promoting their motility and invasion (Takuwa, 2002; Visentin *et al*, 2006). Moreover, cells transfected with SPHK can produce tumours when orthotopically xenografted into nude mice, and the resultant tumours are resistant to cytotoxic chemotherapeutics (Pchejetski *et al*, 2005). These results are consistent with a study showing that a small-molecule inhibitor of SPHK given i.p can reduce tumour volumes in SCID mice receiving s.c. injections of JC mammary adenocarcinoma cells (French *et al*, 2003). Significantly, the concept that *Sphk1* can be a novel oncogene is further supported by the finding that SPHK1 is overexpressed in many human solid tumours, such as those of the breast, colon, lung, ovary, stomach, uterus, kidney and rectum (French *et al*, 2003; Johnson *et al*, 2005; Kawamori *et al*, 2006). As a consequence, the increase in SPHK1 expression in tumour biopsies has been correlated with a significant decrease in survival rate in patients with glioblastoma multiforme (Van Brocklyn *et al*, 2005).

Importantly, it has been demonstrated that several human tumour-derived cell lines become apoptotic when treated with SPHK small-molecule inhibitors, and that their effectiveness can be accounted for by their abilities to reduce S1P levels (Bektas *et al*, 2005a). Similarly, downregulation of the kinase by siRNA decreases resistance of melanoma cells to apoptosis, whereas the protective effect of enhanced Bcl-2 expression has been attributed to increased SPHK expression (Bektas *et al*, 2005a).

Taken together, these findings demonstrate an important concept that S1P is a tumorigenic growth factor likely produced by tumour cells themselves and that lowering the concentration of S1P may be useful in anticancer therapy.

## SPHINGOSINE-1-PHOSPHATE AND TUMOUR ANGIOGENESIS

Angiogenesis is the process by which new blood vessels are formed from existing vasculature. The angiogenesis process associated with tumours (tumour angiogenesis) is considered to be a crucial component of disease progression. Antiangiogenic therapeutics is particularly attractive because vascular endothelial cells (ECs) do not mutate as easily as do cancer cells; consequently, ECs are less likely than cancer cells to gain resistance to prolonged therapy, making them good potential targets for therapeutics. The antiangiogenic approach to cancer has been greatly advanced by the recent FDA approval of the antiangiogenic drug, bevacizumab (Avastin®, Genentech, South San Francisco, CA, USA) to treat colon cancer as an adjunct to cytotoxic chemotherapy.

A growing body of recent evidence implicating S1P as one of the most potent proangiogenic agents comes from studies directly comparing S1P with agents such as VEGF and bFGF. Sphingosine-1-phosphate stimulates DNA synthesis and chemotactic motility of human venous ECs (HUVECs), whereas inducing differentiation of multicellular structures, all of which is suggestive of S1P's role in early blood-vessel formation (Lee *et al*, 1999; Liu *et al*, 2000; Argraves *et al*, 2004). Also, S1P promotes the migration of bone marrow-derived EC precursors to neovascularisation sites (Annabi *et al*, 2003). Cells that overexpress S1P_1_ are resistant to the antiangiogenic agents thalidomide and Neovastat (Annabi *et al*, 2003). In addition, it has been demonstrated that substantial cross-talk exists between S1P and other proangiogenic growth factors such as VEGF, EGF, PDGF, bFGF and IL-8. For example, S1P transactivates EGF (Shida *et al*, 2004) and VEGF2 receptors (Spiegel and Milstien, 2003) and VEGF upregulates S1P_1_ receptor expression (Igarashi *et al*, 2003). Also, S1P, acting via S1P_1_ and the ‘VEFG axis,’ is required for hind-limb angiogenesis and neovascularisation (Chae *et al*, 2004).

The most direct *in vivo* evidence that S1P contributes to tumour angiogenesis comes from our recent publication that focused on a murine monoclonal antibody (mAb) designed to neutralise extracellular S1P by molecular absorption (Visentin *et al*, 2006). In various *in vitro* assays using HUVECs, the anti-S1P mAb neutralised tube formation, migration of vascular ECs and protection from cell death, each of which is S1P-induced. Sphingosine-1-phosphate increased new capillary growth into Matrigel plugs implanted in mice, an effect that was neutralised by the systemic administration of the anti-S1P mAb. The mAb substantially neutralised bFGF- and VEGF-induced angiogenesis in a murine Matrigel plug assay, and the antibody mitigated S1P stimulated the release of proangiogenic cytokines (VEGF, IL-8 and IL-6) from tumour cells *in vitro* and *in vivo*. Importantly, mice xenografted with orthotopically placed human cancer cells exhibited substantial retardation of tumour progression with anti-S1P mAb treatment. This was demonstrated in murine models of human breast, ovarian and lung cancer and in one allograft model of murine melanoma (Visentin *et al*, 2006).

## TARGETING S1P AS A LIPIDOMIC-BASED THERAPEUTIC INTERVENTION

It is now well accepted to target the protein ligand of a receptor (e.g., Avastin targets VEGF; Remicade and Humira target TNF*α*) for the purpose of intervening in the signalling cascade that would otherwise have been activated by the ligand–receptor interaction. Monoclonal antibodies like Avastin have been particularly useful as molecular sponges to selectively absorb and neutralise the protein ligands that would otherwise activate receptors.

The anti-S1P mAb is potentially valuable in that it is a reagent that targets a bioactive-lipid ligand, in this case S1P. By comparison with large protein ligands like VEGF (35 000–44 000 Da), the lipid target, S1P, is a small molecule (379 Da) and, as a consequence, has only one epitope (i.e., the polar head group) to target for antibody recognition (inset of Figure 1). Unlike the successful work with anti-PGE2 mAbs (Portanova *et al*, 1996), academics and industry researchers have not been successful in developing mAbs that inhibit bioactive lysolipids like S1P and LPA with antibody specificity, affinity and other performance characteristics suitable for preclinical proof-of-concept work. This leads one to argue that sphingomab may be a drug of an emerging discipline that may be termed lipidomic-based therapeutics.

With a few notable exceptions (e.g., folic acid targeted by methotrexate), the vast majority of the >500 molecular drug targets are proteins. Second to protein targets are nucleic acids, accounting for about 2% of the total targets. The focus on proteins was a natural consequence of our evolving understanding of biochemistry, which allowed researchers to identify potential protein targets involved in key metabolic and signalling pathways. Some of the first drugs developed by the rational drug design approach to the scientific method came after the discovery of key enzymes, receptors and ion channels as they emerged in the basic science literature. One can argue that target identification now is driven by the technological developments of proteomics and genomics, both of which reflect our persistent ‘protein-centric’ view of drug discovery.

Now, the field of lipidomics (a subset of ‘metabolomics’) has emerged (Wenk, 2005) and provides new opportunities for drug discovery. As was the case for proteomics and genomics, tools of measurement led the way. For lipidomics, the development of electrospray tandem mass spectrometry and other tools has facilitated our understanding of the cellular lipidome, and we now believe that there are over 1000 members of the lipidome, opening up an entire array of new potential targets for therapeutic interventions.

It has been recognised that alterations in lipid metabolism can lead to cancer, cardiovascular disease, diabetes, neurodegenerative disorders, immune function, pain, mental disorders and inflammation. However, only as a consequence of our recent appreciation that bioactive lipids are *bona fide* signalling molecules have key members of the functional lipidome been viewed as targets for rational drug design. The best example of signalling lipids to target might be prostaglandins as proinflammatory agents. The Cox-2 inhibitors designed to mitigate prostaglandin production not only are effective NSAIDs but are now proving useful in clinical trials for cancer therapy.

Of late, the attractiveness of the sphingolipid group of bioactive lipids as targets has been fuelled by the recent discovery, beginning largely in the mid-1990s, that many small-molecule lipid mediators are ligands for GPCRs (e.g., S1P and LPA on EDG receptors), ion channels (e.g., SPC action on SCaMPER) and/or modulators of key kinases (e.g., SPH action on PKC) and transcription factors (e.g., LPA action on PPAR*γ*).

When searching the functional lipidome for new anticancer targets, researchers with a protein-centric view focus on the lipid proteome, rather than on the lipidome itself. As such, in the antisphingolipid-therapeutics arena, several pharmaceutical and biotechnology companies have chosen the classical approach of targeting enzymes of the sphingolipid biosynthetic pathway, such as SPHK1, or one or more of the five GPCRs identified for S1P (S1P_1−5_).

Unfortunately, targeting SPHK is somewhat complicated by the finding that SPHK1 and SPHK2 may have opposite actions (Maceyka *et al*, 2005a). Sphingosine kinase1 is thought to be translocated to the surface membrane and to produce/release the extracellular S1P that is tumorigenic and proangiogenic. Thus, SPHK1 is thought to produce the extracellular S1P that protects cancer cells from proapoptotic cytotoxics (Figure 2). On the other hand, SPHK2 induces apoptosis and inhibits growth (Maceyka *et al*, 2005b). The SPHK2 is likely to produce S1P exclusively for intracellular signalling.

Because of the divergent roles of the two SPHK isoforms, a SPHK-targeted anticancer therapeutic would have to selectively inhibit SPHK1 and not SPHK2. This may prove to be difficult, as both enzymes have five highly conserved domains and the resulting high-percent identity in their amino-acid sequences. In addition, several splice variants of both isoforms have been characterised and a third isoform of SPHK has been recently identified (Bektas *et al*, 2005b), thus further complicating target selectivity. Novagen's anticancer genestein derivative, phenoxodiol, currently in Phase 3 trials, is thought to have some activity as an inhibitor of SPHK (Gamble *et al*, 2006).

Other SPHK-independent sources of S1P have been suggested. One such player is the exoenzyme, autotaxin, which is capable of producing S1P from SPC (Clair *et al*, 2003). Potential additional sources of S1P like the autotaxin story may explain why *sphk1*-null mice exhibited no significant changes in tissue S1P levels even though tissue SPHK activity was nearly eliminated (Allende *et al*, 2004). However, autotaxin KO mice exhibited no significant changes in S1P levels either (van Meeteren *et al*, 2006); so, it remains unclear as to how dominant SPHK is as the sole source of tissue or blood S1P.

Another classical approach to intervening in the sphingolipid pathway is preventing S1P from interacting with its compliment of S1PRs. The potential success of this strategy has been simulated by studies showing that the antineoplastic and antiangiogenic effect of the sphingolipid analogue, FTY720 (Lamontagne *et al*, 2006) can be attributed to its downregulation of four of the five S1PRs (Cuvillier and Levade, 2003). One complication to the antireceptor approach is that not all S1PRs mediate tumorigenic responses. Although S1P_1_ and S1P_3_ are responsible for the proproliferative, promigratory and antiapoptotic effects of S1P, S1P_2_ has been shown to have the opposite effects on cell migration and other responses (Goparaju *et al*, 2005; Sanchez *et al*, 2005). Thus, as is the case with SPHKs, targeting S1PRs may be complicated by the presence of multiple isoforms with opposing actions on tumour cells, and selectivity of receptor antagonism will be a key element in successful sphingolipid receptor-based therapeutic interventions.

A more direct approach that avoids these limitations is the prevention of ligand binding to all cognate receptors, the approach that could be used by the highly specific mAb to S1P. Preclinical data demonstrate that this approach deprives growing tumour cells of important growth and survival factors and largely mitigates tumour angiogenesis (Visentin *et al*, 2006). Because of the important role of sphingolipids in cancer progression, it has been argued that sphingolipid-based therapeutics will be the next generation of cancer treatments (Milstien and Spiegel, 2006).

## CONCLUSION

The use of the anti-S1P mAb as a research tool has provided strong evidence that S1P has several mechanisms of action in promoting cancer, including: (i) direct effects of S1P on tumour-cell growth and metastatic potential, (ii) direct angiogenic effects on ECs in their tumour angiogenesis roles and (iii) indirect angiogenic effects of S1P in stimulating the release and action of other proangiogenic growth factors such as VEGF (Figure 2). This work, as well as recent strides in our understanding of the cellular lipidome, gives credence to an emerging area of drug discovery called lipidomic-based therapeutics that directly targets pleiotropic bioactive lipids involved in cancer as well as other disorders.
